# Understanding Factors to COVID-19 Vaccine Adoption in Gujarat, India

**DOI:** 10.3390/ijerph19052707

**Published:** 2022-02-25

**Authors:** Viral Tolia, Rajkumar Renin Singh, Sameer Deshpande, Anupama Dave, Raju M. Rathod

**Affiliations:** 1 Post Graduate Department of Business Management, Sardar Patel University, Anand 388120, Gujarat, India; viral.tolia@marwadieducationedu.in (V.T.); rajumrathod@rediffmail.com (R.M.R.); 2 Shanti Business School, Ahmedabad 380058, Gujarat, India; renin@shantibschool.edu.in; 3 Social Marketing @ Griffith, Griffith Business School, Nathan Campus, Griffith University, Brisbane, QLD 4111, Australia; 4 School of Business and Law, Navrachana University, Vadodara 391410, Gujarat, India; anupamad@nuv.ac.in

**Keywords:** COVID-19, vaccine uptake, social marketing, India

## Abstract

The COVID-19 pandemic has posed threats to human life across the globe, including India. Vaccinating is an effective means of addressing the pandemic threat. The government of India has implemented a massive vaccination drive to save its citizens from the deadly virus. However, the effort has faced multiple challenges, including vaccine hesitancy. This research understands respondents’ perspectives on factors contributing to the lower vaccination uptake in Gujarat, India. Forty-four semi-structured interviews were conducted through convenience sampling representing different demographic backgrounds. Factors influencing vaccine adoption included religious leadership, political leadership and the government, and fear of side effects, especially among children and those with co-morbidities, resulting primarily from fake news and misinformation circulated through social media. Compared with nine countries from across the world, the study found similarities to vaccine hesitancy from misinformation and the fear of side effects among children. In contrast, the role of government and the influence of religious and political leaders was considered positive. The study recommends strategies to overcome people’s apprehensions about the adoption of vaccination. These include offering incentives, providing positive peer-to-peer communication, recruiting influencers such as religious and community leaders and early adopters such as the elderly population to endorse vaccination, targeting youth through social media, and reaching rural sections by involving NGOs and social service groups.

## 1. Introduction

The evidence demonstrating the benefits of immunization is overwhelming. It is one of the most significant and cost-effective means to improve health outcomes [1]. Vaccines have reduced the overall morbidity and mortality of several infectious diseases in the past [2]. Vaccination can similarly play a substantial role in controlling the COVID-19 pandemic [3]. Despite these benefits, a strong minority in society remains opposed to vaccines. Such opposition can derail the government’s attempt to protect the citizens and achieve public health objectives. To overcome this problem, it is important to understand the reasons for this opposition. The current study explores perceptions of the COVID-19 vaccine among residents of Gujarat, India.

### 1.1. Vaccine Hesitancy

The Strategic Advisory Group of Experts (SAGE) Working Group defines vaccine hesitancy as the “delay in acceptance or refusal of vaccination despite the availability of vaccination services. Vaccine hesitancy is complex and context-specific, i.e., varying across time, place, and vaccines. It is influenced by factors such as complacency, convenience and confidence” [4] (p. 4163). Vaccine hesitancy lies in a continuum between the two extremes of individual behaviour, i.e., total acceptance and opposition to vaccination [5,6].

The World Health Organization (WHO) highlighted vaccine hesitancy as one of the top global threats [7]. Vaccine hesitancy might be observed in a mere ten per cent of the population [8]. However, they can complicate the vaccination drive as the rest of the population might be influenced by negative word of mouth [9]. Hence, understanding the reasons for the lower uptake of vaccines is critical from policy formulation and social marketing intervention perspectives.

### 1.2. Factors Related to Vaccine Hesitancy

Numerous studies have found that people do not consider vaccines safe and effective, thereby doubting the vaccination program [10,11,12]. This uncertainty has resulted in lower-than-expected vaccination rates, especially in low- and middle-income countries [13,14,15]. The issue is not limited to the developing world. Studies on fear of vaccines, low trust in government, and the role of misinformation have also been reported in developed countries [8,16,17,18,19].

### 1.3. Vaccination Campaign and Status in India

The COVID-19 vaccination program began in India on 16 January 2021. Consistent with the WHO guidelines, the government of India told the supreme court that the COVID-19 vaccination is neither mandated nor related to social services or benefits; it is voluntary for all [20,21]. The Indian government established a National Expert Group on COVID-19 Vaccine Administration (NEGVAC) to advise on all areas of COVID-19 vaccine administration in India [22]. The government formed a group of specialists from various branches to determine the clinical criteria that should be used to prioritize persons with co-morbidities for COVID-19 immunization. According to the committee, anyone with congenital heart disease or any other kind of disease was given top priority. The COVID-19 vaccination was initially administered to healthcare personnel, frontline workers, and those over 50 (with a priority for those over 60), followed by people under 50 with co-morbidities.

The population aged 50 years and above were prioritized for vaccinations in phase one, as morbidity and mortality in this group were the highest compared to other age groups. Identification of the population aged 50 years and above was created based on the latest electoral roll of the general election.

The deadline for calculating age was 1st January 2021. If eligible people were missed from the list, they could provide identity and self-register for vaccination [23,24,25]. Despite several challenges in India (i.e., limited healthcare infrastructure, limited vaccine availability, geographic constraints, and a large population), the country had administered both vaccination doses to 461.5 million people 18 years and above (49%) as of 31st July 2021. On the same day, Gujarat had administered two doses to 32.6 million people 18 years and above (66%) [26,27,28,29,30].

### 1.4. Setting

The research area selected is Gujarat, located in the western part of India, with a projected population of approximately 70 million in 2021 and comprising 907 women per 1000 men [31]. The literacy rate in 2011 was 78% [32], which is expected to have increased in the past decade. While the people of Gujarat mostly follow Hindu practices, a sizeable population adheres to Islam, Christianity, Sikhism, Buddhism, and Jainism. This state has an average healthcare facility with over 23 district hospitals, 8347 rural dispensaries, 14 homeopathic hospitals, 1046 ayurvedic dispensaries and 48 ayurvedic hospitals [31].

During COVID times, it was observed that the Gujarati community was keener to consume ayurvedic medicine over Western medicines; around 77% in India and 53% in Gujarat prefer ayurvedic medicines. Besides this, it was observed that instead of taking vaccines, the Gujarati population preferred homemade remedies [33,34].

Due to the low vaccination rate, vaccine hesitancy, and preference for home remedies, researchers decided to explore factors of COVID-19 vaccination in Gujarat.

Implementing a social marketing strategy to curb vaccine apprehensiveness is vital [35]. As per social marketing research, one-time behaviour change is easier to promote, and vaccination falls into this behaviour category [36,37,38]. There is a need to develop consciousness among the people and a positive intention towards receiving the vaccines so that the Gujarati population receives the required doses of COVID-19 vaccination. In this state, vaccination shots are available in different inoculation centers [39] at a low price [40].

In addition to analyzing the perceptions of Gujarati residents on vaccination, the current study proposes strategies from a social marketing lens to help the government of India effectively reduce the skepticism concerning the vaccines among the mass and increase the uptake of COVID-19 vaccine shots. This study contributes to the social marketing literature by discovering themes related to the lower uptake of vaccines in the context of low-income but rapidly developing countries.

The paper has been structured in the following manner: First, we describe the method used in the study. Second, we present the themes emerging from the study. Third, we compare the themes with those found in studies in other countries. Further, the social marketing strategy for the government has been explored in practical implications. Finally, the study’s limitations and conclusion are discussed. This study demonstrates how the lens of social marketing can be applied to increase the uptake of COVID-19 vaccine jabs in Gujarat.

## 2. Method

Semi-structured interviews were administered to identify the perceptions towards the COVID-19 vaccine from respondents residing in Gujarat, India. Morse [41] and Cresswell [42] have suggested that the saturation range in semi-structured interviews is around 5 to 25. However, more interviews were undertaken to achieve saturation [43] and provide an in-depth understanding of a relatively new phenomenon [44].

Prior to collecting data, the study was approved by Sardar Patel University. Sameer Deshpande was not involved in the data collection process, and Griffith university was not involved in the human subjects ethics approval process. The respondents were recruited using purposive sampling, wherein the researchers approached participants representing varied demographic (age, gender, and religion) and geographic segments. Data were collected utilizing face-to-face and digital platforms (Google Meet).

An interview guide was prepared based on the literature on risk communication and social marketing. It was divided into two parts: the first part was related to demographic factors (five questions), and the second part was related to perceptions of the COVID-19 vaccine (two questions each related to general vaccines, lower uptake, the role of vaccines, government initiative and religion). An expert was consulted to validate and finalize the interview questions. The interview guide is provided in Appendix A.

Interviews were conducted and reviewed by two authors, (VT) and (RR), and the process was terminated on data saturation [45]. One of the authors developed transcripts in the first stage. In the second stage, both the coders independently read the transcripts and assigned the codes to relevant quotes. In the third stage, the authors merged the relevant codes into sub-themes, and sub-themes into themes in the last stage. At each stage, the coders met and resolved discrepancies; when needed, the third coder was consulted (RMR). Intercoder reliability of 80% was noted, and discussions resolved discrepancies.

Both audio and video conversations were recorded for all the interviews with the prior permission of the respondents. The interviews were conducted on average for 40 min at a location convenient to the participant. Proper care was taken to maintain participant confidentiality at every interview stage (pre, during, and post-interview). Written consent was taken from all the participants before conducting the interview. An open exit option was available to the participants if they desired to quit the interview prematurely. The interview process was entirely voluntary, and no incentive was offered to participate in the interview. The interview data were then transcribed and translated to English (for interviews conducted in Gujarati). The translation was completed by a native speaker and was cross validated by two other native speakers. The data was thematically organized and analyzed with the help of NVivo 12 (QSR International) software [46].

## 3. Results

**Figure 1:**
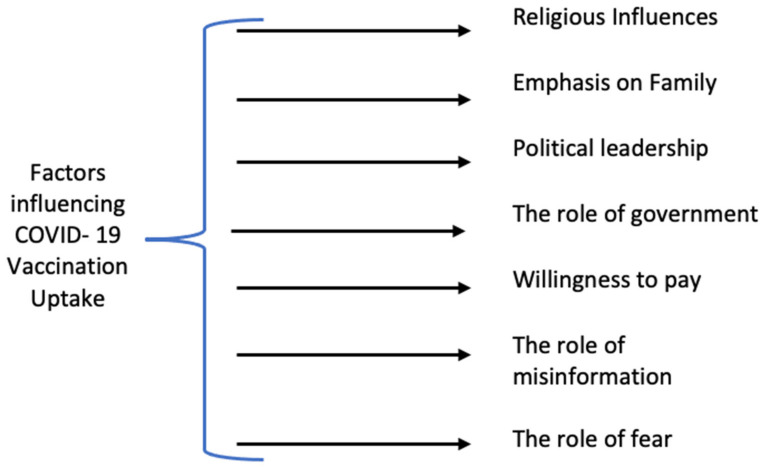
Seven themes affecting vaccination drive in Gujarat.

Overall, 44 interviews were conducted and analyzed. The demographic and geographic details of the respondents are presented in Table 1, who resided in different cities of Gujarat. Seven themes emerged from the interviews (See Figure 1). The obtained themes from primary data were arranged from broader to narrow scope for better organization and ease of interpretation [47]. The themes and subthemes derived from the interviews are discussed below. The themes were later compared with nine select countries (Table 2). A detailed explanation of the comparison with nine countries is presented in Section 4.

### 3.1. Low Uptake of Vaccines

Most participants expressed their views on vaccines and described the factors influencing them.

“I already had corona once. Now, we are safe at least for three to four months. We have antibodies in our body, so we do not need to take the vaccination”.

(participant 7)

“I have heard all this kind of news of vaccine inefficacy. I do have hesitancy and fear. You will not believe it; I have not even taken a test to check whether I have corona or not till today! So, forget about the vaccine”.

(participant 27)

### 3.2. Themes

#### 3.2.1. Religious Influence

Several participants informed that religion has a positive, significant, and long-term influence on their decision to take the COVID-19 vaccine. They refer to religious norms in culture, politics, or economic concerns. The participants accepted that religion influences the lower uptake of vaccines.

“Religion will play a role. See, the thing is that Indians are quite religious. We always bring religious sentiments when eating food or wearing dresses like Western culture dresses. Also, I feel that many people would not prefer to have it (the vaccine) based on religious sentiments”.

(participant 14)

##### Influence of Religious Leaders

Twenty-six participants stated that religious leaders would influence the vaccination process. According to them, religious leaders have the power to influence people in their daily life, irrespective of their lifestyle or educational background. They accentuated that many religious leaders came forward to guide people during the initial lockdown phase in India.

“If religious leaders are educated about the vaccination process, they will educate the local people or their community and explain to them the importance of vaccine”.

(participant 18)

##### Ingredients of Vaccine and Religious Sentiments

One of the followers of Islam raised the aspect of halal (permissible) in vaccination but clarified that it would not alter their decision related to vaccination. The content of the vaccine, whether halal or haram (impermissible), is irrelevant to them, and they would prefer to take the vaccine.

“When it comes to vaccine ingredients, it will not matter to me what is inside the dose. For example, we are given medicine like cough syrup when we have an illness. It contains alcohol (which is haram), but we overlook this aspect. We take medicines regularly, so we cannot claim that we have not taken alcohol. I will take the vaccine as vaccine and avoid thinking about this aspect”.

(participant 42)

The other follower of Islam had an opposite stand. They would refuse to take the vaccine if it did not follow Islamic laws.

“The main reason I am apprehensive about the vaccine is that I do not know exactly what is in the vaccine. I will accept it only if the ingredients are halal (and not haram)”.

(participant 6)

#### 3.2.2. Emphasis on Family

##### Children

Almost all the participants were hesitant to vaccinate their young children. The reasons are (a) vaccines are not tested for children yet, (b) usually, children are at home, (c) they would like to consult the doctor first, and (d) the vaccine might have an adverse reaction on them.

“When it comes to children, one has to be very careful as they have a long life ahead. It will be a big problem if some major side effect happens. The first thing is to consult a doctor, make sure that they are mentally and physically capable of taking the vaccines, only then the vaccine should be given to them”.

(participant 25)

##### Seniors

Several participants said that they prefer the old-aged members in their family to be vaccinated. Low immunity in old-aged people makes them susceptible to the virus, and the vaccine is vital to fight against COVID-19—however, a few recommended caution due to potential side effects.

“Yes, elders should go for vaccination because their immune system is not strong. Also, many of them have other diseases and co-morbidities. The doctor’s advice is vital before taking the vaccine in such cases”.

(participant 14)

“I feel like old people should go for the vaccination, but if they have some other major disease like high blood pressure, diabetes or cancer, then they need to think twice, and for the same, they should take the advice from their family doctor first”.

(participant 17)

##### Young People

Almost everyone posits that youth should be vaccinated as early as possible. Reasons stated were (a) high level of exposure either due to personal or professional work and (b) fear related to spreading COVID-19 disease during the pandemic.

“Young people should go for vaccination because they are the main sources of spreading. Since they travel to different places for professional and personal reasons, they meet many people who might be the carriers of the virus”.

(participant 19)

#### 3.2.3. Political Leadership

Almost all the participants said that the role of political leaders is extremely influential. Respondents remarked that they were greatly influenced by the prime minister of India (Mr. Narendra Modi) and other political leaders who took the vaccine.

“Our honorable prime minister of India, as well as the chief minister of Delhi, have taken the vaccine, and hence people are influenced to follow suit. People felt assured that they took vaccinations as they were certain about their effects. Thus, personally taking the vaccine and suggesting others do the same will significantly influence creating a positive attitude towards the vaccine. Celebrities and politicians generally have a strong influence on people”.

(participant 8)

#### 3.2.4. The Role of Government

Most participants vocalized that the government has taken sufficient measures concerning COVID-19 vaccination. Furthermore, they believed that the government had utilized the available resources effectively to develop the entirety of the vaccination program. Appreciation was also expressed concerning the safe and hygienic delivery of the vaccination to the public.

“Yes, the government (across India) has undertaken sufficient initiatives to guide people; they have created awareness about vaccines using local languages like Gujarati, Hindi, Marathi and that too in layman’s terms. They have even put a caller tune of around 30 s to spread awareness about taking vaccines when the slots are available. Sometimes it was irritating, but they have used it positively to influence people by delivering necessary information”.

(participant 12)

“From Rajkot municipality corporation, a team of three people including a doctor used to come to our home at a regular interval to check about our health condition. They used to bring the COVID test kit, and if found positive, they would provide free basic medicine”.

(participant 40)

“As my daughter was found COVID positive, she returned for a few days from her in-law’s family. She told me, every Tuesday and Friday, she used to get a call from the local municipality to check about her health”.

(participant 37)

Participants also suggested the following ideas to improve the vaccination uptake:

(a) to control the spread of misinformation both online and offline
(b) to provide vital information to the masses, especially to those located in rural areas and the weaker sections
(c) to increase the supply of vaccines to private hospitals for easy access
(d) to provide data of state-wise measures taken in the country
(e) to ensure transparency concerning the vaccination process
(f) to provide vaccines to private corporate employees as a priority
(g) to collaborate with NGOs and other service provider organizations, and
(h) to bring transparency by sharing vaccination data

#### 3.2.5. Willingness to Pay

Nearly all participants said that they were willing to pay for the vaccine. The principal reason behind it was that they trust private health institutes more than government health institutes. The pretext for the same is (a) hygiene and safety, (b) affordable price of the vaccine, (c) reluctance towards government vaccination centers, (d) not to burden the government and (e) help a needy person instead.

“I paid for my parents’ vaccination. My reason was not safety or hygiene or anything like that. My reason was that I did not want to burden the government with additional 500 rupees”.

(participant 28)

“If I am supposed to take the vaccine, I will pay for it because I can afford it. US $7 for two shots is fine. The genuine reason for not going to the government health center is that if I give up my vaccine shot, a needy person can get that shot, and they will not have to pay for it. As I can afford it, so I can get it from a private hospital”.

(participant 43)

#### 3.2.6. The Role of Misinformation

The participants also stated that they come across fake news or misinformation about vaccines’ safety and side effects, especially on social media platforms like Facebook and WhatsApp.

“There are stories related to vaccine floating around saying that if we take the vaccine, then our genetic makeup changes. We are surrounded by a lot of misinformation about the vaccine because we do not have 100% information related to the vaccine”.

(participant 30)

#### 3.2.7. The Role of Fear

The role of fear in low vaccine uptake was investigated among the respondents. Fear among the respondents was related to (1) side effects of vaccines and (2) the vaccine trial process.

“My grandmother and my mother both have blood pressure and diabetics. I insisted they go for vaccination, but they were concerned about what would happen after taking the vaccine. That is why they were hesitant”.

(participant 15)

“I am afraid because as we are young. I do not think corona will easily enter our body if we take care. Because of the lockdown, we have already improved our health. So, I do not feel the need for the vaccine because it may instead cause damage”.

(participant 4)

Many participants highlighted that they would like to cure themselves through ayurvedic medicines instead of vaccine jabs for three reasons: first, it is mainly prepared with homemade ingredients; second, they are comparatively easily accessible; and third, they have fewer side effects.

“When I have a health problem, I choose Ayurveda medicine since my brother’s wife, brother-in-law, and father-in-law are all ayurvedic physicians. Only ayurvedic medicines are used to treat illnesses in our family, and I am fearful of other drugs”.

(participant 7)

## 4. How Do Our Findings Compare with the Rest of the World?

Vaccination hesitancy has been a matter of concern across the world. This section compares perceptions to COVID-19 vaccination of this research with studies from select countries: the USA, Germany, Canada, the UK, Australia, Brazil, Saudi Arabia, Japan, and Thailand. These countries were selected because they: (a) collectively represent most continents; (b) have each administered, by 27 November 2021, at least one jab of the COVID-19 vaccine to 40% of the eligible population [48]; and (c) collectively represent most major religions (Christianity practiced in USA, Germany, Canada, UK, Australia, and Brazil, Islam in Saudi Arabia, Shintōism in Japan, Hinduism in India, and Buddhism in Thailand) [111], This diversity helped us examine the cross-cultural effect and its influence at the global level [112,113,114]. A comparison is represented in Table 2**.**

Religion had a significant effect in Australia in relation to our first theme. Edwards, Biddle, Gray and Sollis [8] reported that very religious people tend to be more resistant to vaccine uptake. Consistently, Smith, Attwell and Evers [51] found that less religious people were more supportive towards uptake. In Japan, Lahav, Shahrabani, Rosenboim and Tsutsui [56] also revealed that more religious people were less likely to take vaccines than less religious ones. In the other developed countries like the USA and the UK, change in religious belief was reported to significantly correlate with the impact of the coronavirus crisis [49].

In the HPV vaccine context, Wong, Wong, Megat Hashim, Han, Lin, Hu, Zhao and Zimet [52] concluded that hesitancy in Muslim-dominated Asian countries exists due to the content of vaccines being haram. This reason could be extended to the COVID-19 vaccine as well. People have stated the same reason in other Muslim countries to refuse the vaccine [53,54]. Our study partially supports this argument.

To overcome these reasons, governments and community groups have employed various tactics. Religious and philosophical exemptions to vaccine laws were recently repealed in Maine, the north-easternmost state in the USA, owing to which 71.6% of Maine is now fully vaccinated compared with 58.9% of the USA population as of 18 November 2021 [50]. The British Islamic Medical Association and other Islamic scholars approved the Pfizer-BioNTech vaccine [62]. While in Thailand, the Sheikul Islam Office has allowed the commencement of mosque prayers in localities where at least 70% of persons aged 18 and above were vaccinated [55].

Analysis of studies from nine countries revealed that family, friends, and other networks influenced vaccinating. For instance, in a survey conducted in the USA by Khubchandani, Sharma, Price, Wiblishauser, Sharma and Webb [57], the family was an influencing factor. Lazarus, Wyka, Rauh, Rabin, Ratzan, Gostin, Larson and El-Mohandes [59] observed that women were more likely than males to follow an employer’s vaccine suggestion in Brazil and the USA. In Canada [58], 70% of university students preferred to take the vaccine only if a doctor or pharmacist recommended it. At the same time, a few instead waited to see the effect of the vaccine on others.

In Japan, vaccines were considered a preventable tool to fight the deadly virus for themselves and their family members [19]. Research conducted among the residents and expatriates in Thailand established that 58% of residents and 82.9% of expatriates would depend on their family member’s advice on the uptake of vaccines [88]. This emphasis on family and doctors resembles the current study with a sample of the Gujarati population.

The third theme that emerged from our study was related to political leaders. While comparing this theme, several similarities were found with other countries. To increase the uptake of vaccines, political leaders across the globe have undertaken several initiatives. For example, thanks to the prime minister’s COVID-19 Vaccine and Treatment Strategy, Australia secured access to four COVID-19 vaccines and over 134 million shots [63]. In Saudi Arabia, the crown prince took the coronavirus vaccine. These government and political leader initiatives were highly appreciated [68]. In contrast, the Brazilian president was criticized for his inability to handle the pandemic [64,65,66,67].

A lack of trust in government and pharmaceutical companies is found in several countries. For example, in Germany, it was found that 58% of people were hesitant towards taking vaccines due to these reasons [71]. Enhancing the public’s trust in the COVID-19 vaccine has been the motto of all governments across the globe. The fourth theme highlighted the government’s active role in promoting the uptake of vaccines. Few efforts were highly appreciated. For instance, the Australian government developed a systematic four-stage process [73] and allocated $257 million to vaccinate 80% of adults by December 2021 [74]. Additionally, the government proactively curbed various companies’ false COVID-19 cure claims by issuing an advisory [75,76].

Furthermore, in the UK, to increase vaccine uptake among the country’s most vulnerable, 16 renowned health charities teamed up with the government and National Health Service to increase the uptake of vaccines [115]. The government started four new programmes in Canada to promote vaccine uptake through the Immunization Partnership fund [72]. In Japan, the government imported 28 million doses by April 2021 [69], significantly improving vaccination rates [82].

Under its National Vaccine Committee, Thailand allocated three billion baht to procure sufficient vaccine supplies [80]. In addition, the Thai government developed an “Anti-fake news centre” to arrest all those who spread false information about the COVID-19 and impose fines [81]. In Saudi Arabia, devotees could only visit two holy mosques for prayers if they had received both vaccine shots [79]. Such initiatives resulted in positive government perceptions [78]. In contrast, the Brazilian government was criticized for their suboptimal performance [48,77].

When political motives influenced vaccine hesitancy, governments responded strongly. In the USA, 29% of Republican supporters have shown hesitancy towards the uptake of vaccines [57]. Some governors have announced mandatory vaccination for state employees [70]. In Canada, 32.4% of respondents argued that vaccines were not evaluated thoroughly and had a political objective [60]. To counter, it was announced that those who refused to divulge their vaccination status or be fully vaccinated would be placed on administrative leave without pay commencing 15 November 2021 [15]. In the UK, England’s chief medical officer urged every woman planning for pregnancy to get her jab in advance as a precautionary measure [61].

These findings resonate with the current study in India.

Willingness to pay is a monetary indicator of a customer’s willingness to pay for a product or service. There are mainly two factors that are connected to the willingness to pay. The first is the type of vaccine, and the second is related to the severity of illness one will face in the future [116]. In Australia, a study revealed that willingness to pay is $34.44 for reducing the waiting time for the uptake of vaccines [87]. According to a study conducted in the USA and Canada, willingness to pay was $23 and $11.5, respectively [83]. In a more recent study, it was found that in the USA, willingness to pay was $228–$291 for a vaccine for themselves and US$243–US$321 for their children [84].

On the contrary, payments did not affect COVID vaccination intentions in Germany [86]. In Brazil, the willingness to pay for vaccine uptake was US$ 22.18 [85], although 75% of people were urged to take the vaccines if they were freely available to them [88]. Our research also found that Gujaratis were willing to pay for their vaccinations, although the value was not ascertained.

There is a thin line between information and misinformation, and especially during the pandemic, misinformation has spread worldwide [117]. Failure to prevent the dissemination of misinformation about COVID-19 and vaccines has caused panic, terror, and disorder in society [118]. In Australia, Rozbroj, Lyons and Lucke [94] concluded that people were highly concerned about the anti-vaccination movement, spreading much misinformation. Between October 2020 and March 2021, TikTok banned 873 videos that mentioned the coronavirus or other medical conditions to stem misinformation [95].

Additionally, Pickles, Cvejic, Nickel, Copp, Bonner, Leask, Ayre, Batcup, Cornell, Dakin, Dodd, Isautier and McCaffery [96] revealed that misinformation was linked with digital health literacy. Evanega, Lynas, Adams, Smolenyak and Insights [89] found that only 16.4% of people did “fact-checking” before passing information to others in the USA. In the UK and USA, through a randomized controlled trial, it was contended that there was a decline in the misinformation by 6.2% in the UK and 6.4% in the USA among those who were open to taking the vaccines [90]. Desveaux, Savage, Tadrous, Kithulegoda, Thai, Stall and Ivers [91] reported that Canadians rely more on public health websites and health care workers’ advice to uptake vaccines.

Through emergency surveillance in Japan, the government targeted companies promoting products that were not permitted and spreading misinformation [76]. A study conducted by Nomura, Eguchi, Yoneoka, Kawashima, Tanoue, Murakami, Sakamoto, Maruyama-Sakurai, Gilmour, Shi, Kunishima, Kaneko, Adachi, Shimada, Yamamoto and Miyata [100] found that people trust physicians and nurses for checking information related to vaccine uptake. A very different approach was adopted in the UK to combat misinformation. The government collaborated with the University of Cambridge to create “Go Viral!”. It was a game developed to teach and enlighten people about how misinformation was being spread on social media to safeguard themselves [92,93].

In Brazil, Coletiva.net [97] found that more than 70% reported the primary source of misinformation to be the WhatsApp application. Similar results were observed in Saudi Arabia, where most misinformation is spread through WhatsApp. Of that, 46% was related to the pandemic [98]. Because of the widespread misinformation in Thailand, Mongkhon, Ruengorn, Awiphan, Thavorn, Hutton, Wongpakaran, Wongpakaran and Nochaiwong [99] revealed that those exposed for three or more hours in a day to misinformation were facing problems related to depression, anxiety, and insomnia.

These perceptions were also observed in our research. Participants relied on medical staff or doctors for vaccine uptake and agreed that various social media tools were reasons for the widespread misinformation.

‘Fear’ is the most significant barrier in the vaccine uptake, which was the final theme in our research. This barrier needs immediate action because it could have a devastating impact if not controlled. While comparing our research results with other countries, some similarities and differences were observed. In Australia, Rhodes, Hoq, Measey and Danchin [105] found 82.8% of people feared vaccine efficacy and safety. Furthermore, a study indicated that one-third of people prefer not to go for the vaccine because they fear the side effects of vaccines [106]. The stress level of people had increased from 10.6% in 2020 to 12.5% in 2021 because of the fear related to vaccination [107]. In Germany, the survey conducted by Bauernfeind, Hitzenbichler, Huppertz, Zeman, Koller, Schmidt, Plentz, Bauswein, Mohr and Salzberger [103] has revealed that 79% of people have fear related to the uptake of the vaccines.

People were hesitant and reluctant (19.9%) to take vaccinations in Japan because of fear of vaccination [19]. Another Japanese study [100] reported similar findings. Furthermore, a recent study by Okubo, Yoshioka, Ohfuji, Matsuo and Tabuchi [110] found that among the hesitant group, more than 70% of people are afraid of the efficacy of the vaccine. In addition to that, Yoda and Katsuyama [19] found that fear resulted from a lack of trust and potential side effects of vaccines. Similar findings were found in the UK [104].

A study in the USA highlighted that 46.2% of people were afraid of vaccine uptake [101]. Kirzinger, Kearney, Hamel and Brodie [102] reported that 48% of Americans had not taken vaccines due to fear, even when health care workers were in direct contact with patients. In Canada, people have shown similar concern for vaccine safety, and because of fear, they were avoiding vaccines [58,60]

Fear from adverse side effects of the vaccine was found among 67.1% in Brazil [108], 35.2% in Thailand [52], and 61.4% in Saudi Araba [54]. These perceptions are similar to the results of this research undertaken for the Gujarati population.

## 5. Discussion, a Review of Government Efforts, and Recommendations

The research goal was to find the real hurdles for the uptake of vaccines in the Gujarati community. The results were categorized into seven themes. Social marketing promotes voluntary behaviour change through user-centric interventions [119,120]. Effective social marketing strategies can help promote ‘one-time behavioral change’ such as vaccine jabs [36,121]. In this section, we consider the social marketing perspective to review the efforts of the governments of Gujarat and India, connect them with the study findings, and recommend strategies to improve vaccine uptake.

The government of India aims to increase the uptake of vaccines in India (short-term) and thereby reduce COVID-19 influence in the long run [122]. Most participants agreed that low vaccination rates threatened the achievement of the long-term goal.

For any campaign to be successful, proper segmentation must be implemented. The Indian government executed this step successfully by implementing a vaccination drive in different phases. The phase-wise execution of vaccination helped them gauge the trust of the younger generation, i.e., 18–35 (the highest ratio of the total population in India), who were the last to get vaccinated.

During the initial phases of the drive, the vaccination was available quickly, which was not the case in the later stage when people above 18 years of age were to be vaccinated. Few participants in the interview highlighted the gap in supply and demand of vaccination. Respondents agreed that the cost of the vaccination was affordable to them, and many were even ready to pay and purchase the vaccine jabs. They preferred to get vaccinated at private hospitals than government centers. The result about the preference for the place is vital, as this is where the expected behaviour would occur and lead to the achievement of short-term and long-term goals.

The government used various promotional strategies to promote vaccine uptake. A few interviewees recalled strategies such as caller-tunes to remind them to wear masks, maintain social distance, and vaccinate. They reported seeing and hearing these messages in Gujarati newspapers, radio, television, flyers, and posters distributed by the local municipality government department.

### Practical Implications

The study offers practical implications to reduce vaccine hesitancy.

This study indicates the need for a social marketing intervention to reduce the hesitancy and increase the uptake of the COVID-19 vaccines. We provide a few recommendations.

One of the most significant barriers is accessibility, as government centers are frequently non-operational. The government department can partner with private clinics and renovate primary health clinics to deliver vaccinations.

Offering incentives can increase the uptake of vaccines. For example, in the USA, the Alabama Department of Public Health sponsored a TikTok Contest for people between 13 to 29 years to post a video of getting vaccinated. Four winners were awarded a $250 Visa gift card. In New York, free lottery scratch-off tickets were given to people 18 years and above with a grand prize of $5 million; such incentives spread positivity about the vaccines [123]

In rural and urban areas, different strategies should be implemented. While traditional and digital media work well in urban India, traditional community media are better suited to reach the target audience in rural India through local street drama (nukkad nataks) and folk dance, to increase awareness about the uptake of COVID-19 vaccines [124].

Government can learn from previous efforts and bust myths to remove negativity. The Vaccines Bring Us Closer campaign during the World Immunisation Week 2021 is an excellent example of showing immunization as a societal norm, asking people from all walks of life to report how vaccines have improved their lives [121]. The “My why” social campaign motivated people to post their stories related to vaccination in Canada on social media. This campaign helped in increasing the confidence of others towards the vaccination. The campaign aimed to increase the overall uptake among Canadians [125]. The Indian government has introduced the “Jan Andolan” campaign. One takes a pledge on a website that he or she will follow six significant behaviors to help safeguard from the deadly virus and receive a certificate [126].

A variety of change agents should be recruited that provide different skills and reach diverse audiences. These could be doctors and frontline workers who influence people by making them aware of the facts of COVID-19 and the efficacy of vaccination and addressing any queries and confusion at a personal level. The next could be parents, who motivate the young generation to vaccinate. Film and sports celebrities would be important to popularize the importance of vaccination through mass media. Additionally, vaccination teams should reach out through phone calls, mobile SMS, and targeted ads on social media sites such as Facebook and employ direct marketing strategies.

Public places such as restaurants, malls, bus and train stations, and cinema halls should be made available to circulate communication materials in vernacular language, in addition to digital and traditional print and audio-visual media. Such forms of communication can remind the unvaccinated or those eligible for a second dose.

Using the internet and social media to increase vaccine uptake is particularly appropriate in Gujarat. According to a recent survey, Surat, Vadodara, and Rajkot have over 57 per cent users, higher than other parts of India [127]. According to the Telecom Regulatory Authority of India (TRAI), Gujarat is currently one of the eight Indian states with a teledensity of more than 100%. Further, mobile phone users have risen in recent months [128].

Children could be reached in schools and taught the importance of vaccines. Specific groups like the Rotary Club, Jain social groups, and mosques can organize camps in their premises to increase the vaccine’s uptake. Corporate leaders can arrange seminars by inviting doctors to address employees who have not yet taken the vaccine.

Even past Indian experiences can help to increase vaccination uptake. For instance, to increase the uptake of polio vaccines, in multiple rounds, medical interns and health care workers visited the residences of Muslims who were resistant to the uptake of polio vaccines and convinced them. Such strategies can be adopted during the COVID-19 pandemic [129].

## 6. Limitations and Future Research

This study has limitations that future research can address. The sample was skewed towards highly educated teaching staff, which does not truly represent India’s educational and occupational profile. This review did not consider the influence of factors like storage of vaccines, transport-related issues, the requirement of booster shots, and the effect of branding of vaccines on its uptake. Another limiting factor is geography; this study was restricted to the state of Gujarat, India. To overcome these gaps, future research should investigate the study themes among respondents of lower-income and non-teaching occupations in and beyond Gujarat.

Additionally, future research should conduct surveys with a larger sample in Gujarat and other Indian states to get a more representative understanding and assess with quantitative and mathematical models. Lastly, the review of studies from the nine countries was driven by the seven themes of the current study. A more grounded approach to exhaustively uncover all perceptions would do justice to consumer insights.

## 7. Conclusions

This research study sheds light on factors that influence vaccine adoption, especially issues relating to lower uptake of vaccines, one of the long-standing problems in the vaccination process. Furthermore, the emerging themes can help develop strategies for social marketers, researchers, and policymakers to promote vaccine acceptance.

## Figures and Tables

**Table 1:** Demographics of participants (n = 44).

Gender	Male	21 (48%)
	Female	23 (52%)
Age	18–25 years	9 (21%)
	26–35 years	16 (36%)
	36–45 years	14 (32%)
	46–55 years	4 (9%)
	56–65 years	1 (2%)
Religion	Hindu	37 (84%)
	Muslims	2 (5%)
	Christian	2 (5%)
	Jain	1 (2%)
	Not Stated	2 (5%)
Level of education	Less than 10th grade	8 (18%)
	High school	2 (5%)
	Diploma	2 (5%)
	Bachelor	4 (9%)
	Master	19 (43%)
	PhD	9 (20%)
Working Status	Academics: Teaching staff	21 (48%)
	Driver	9 (20%)
	Academics: Non-teaching staff	5 (11%)
	Corporate job	2 (5%)
	Businessperson	2 (5%)
	Coaching class counsellor	1 (2%)
	Coaching class teacher	1 (2%)
	Fashion designer	1 (2%)
	Homemaker	1 (2%)
	Street hawker	1 (2%)
Marital Status	Married	29 (66%)
	Unmarried	13 (30%)
	Prefer not to say	2 (5%)
City in Gujarat	Rajkot	37 (84%)
	Surat	3 (7%)
	Ahmedabad	1 (2%)
	Anand	1 (2%)
	Vadodara (Baroda)	1 (2%)
	Bharuch	1 (2%)

**Table 2:** Country-wise comparison.

	Major Factors	Northern America	Europe			Oceania	Latin America and the Caribbean	Asia			
	Country	USA	Germany	Canada	UK	Australia	Brazil	Saudi Arabia	Thailand	Japan	India (Current Study)
	Eligible population (million) and % of population given at least one dose of vaccine (accessed on 27 November 2021) [48]	200 (65.3%)	60 (76%)	30 (77.9%)	50 (73.1%)	20 (66.4%)	200 (71.6%)	20 (68.2%)	30 (42.3%)	90 (70.6.%)	700 (47.7.%)
1	Religious influence	Rigoli [49], Graves [50]		-	Rigoli [49]	Edwards, Biddle, Gray and Sollis [8], Smith et al. [51]	-	Wong et al. [52], Huda E. Zainudin et al. [53], Padhi and Al-Mohaithef [54]	Pakkawan [55]	Lahav et al. [56]	Yes
2	Emphasis on family members	Khubchandani et al. [57]	-	Mant et al. [58], Lazarus et al. [59]	Lazarus, Wyka, Rauh, Rabin, Ratzan, Gostin, Larson and El-Mohandes [59]	-	Lazarus, Wyka, Rauh, Rabin, Ratzan, Gostin, Larson and El-Mohandes [59]	-	-	Yoda and Katsuyama [19]	Yes
3	Political leadership	Khubchandani, Sharma, Price, Wiblishauser, Sharma and Webb [57]	-	Griffith et al. [60]	BBC news [61], Mirza [62]	Ministers Department of Health [63]	Burki [64], Fonseca et al. [65], Lancet [66] Marcello and Boadle [67]	Reuters Staff [68]	-	Kosaka et al. [69]	Yes
4	The role of government	The White House [70]	Holzmann-Littig et al. [71]	Public Health Agency of Canada [72], [15]	The White House [70]	Australian Government [73], Bennett [74], Australian Government [75], Freckelton Qc [76]	The Tribune [77], Reuters [48]	Al-Mohaithef et al. [78], Ani [79]	Rattanachaikunsopon and Phumkhachorn [80], Namwat et al. [81]	Kosaka, Hashimoto, Ozaki, Tanimoto and Kami [69], Hayes [82]	Yes
5	Willingness to pay	Wong et al. [83], Catma and Reindl [84], Godói et al. [85]	Sprengholz et al. [86]	Wong, Alias, Wong, Lee and AbuBakar [83]		Borriello et al. [87]	Kitro et al. [88]	-	-	-	Yes
6	The role of misinformation	Evanega et al. [89], Loomba et al. [90]	-	Desveaux et al. [91]	Loomba, de Figueiredo, Piatek, de Graaf and Larson [90] Lewsey [92], OECD [93]	Rozbroj et al. [94], Taylor [95], Pickles et al. [96].	Coletiva.net [97]	Alasmari et al. [98]	Mongkhon et al. [99]	Freckelton Qc [76], Nomura et al. [100]	Yes
7	The role of fear	Trent et al. [101], Kirzinger et al. [102]	Bauernfeind et al. [103]	Griffith, Marani and Monkman [60], Mant, Aslemand, Prine and Jaagumägi Holland [58]	Iyengar et al. [104]	Rhodes et al. [105], BBC News [106], The New Indian Express [107], Yoda and Katsuyama [19]	Moore et al. [108]	Padhi and Al-Mohaithef [54]	Thanapluetiwong et al. [109]	Nomura, Eguchi, Yoneoka, Kawashima, Tanoue, Murakami, Sakamoto, Maruyama-Sakurai, Gilmour, Shi, Kunishima, Kaneko, Adachi, Shimada, Yamamoto and Miyata [100], Okubo et al. [110], Yoda and Katsuyama [19]	Yes

## Data Availability

Research data are not shared.

## Appendix A

**Table A1:** Semi-structured interview guide for vaccine hesitancy.

No.	
A	Set of demographics details
B	Set of main questions
1	What are your views about the COVID vaccine?
2	What have you heard about the vaccine from various sources?
3	Do you have any hesitancy or fear regarding taking the vaccine?
4	Have you taken or would you take the vaccine? Why or why not?
5	What about your family members? Will you allow them to take the vaccine? [Emphasize both on children and elders, also try to find if they have co-morbidities]
6	What do you think is the role of religion in the acceptance of the COVID vaccine? For example, the role of religious leaders or halal? Do you know of anyone who has opted not to take the vaccine due to religious reasons?
7	What is your opinion on the effectiveness of the vaccine?
8	What is the role of misinformation in vaccine hesitancy?
9	What steps should be taken be taken to remove misinformation?
10	Do you think the government has taken sufficient measures to communicate the vaccine’s effectiveness?
